# Toward clinical digital phenotyping: a timely opportunity to consider purpose, quality, and safety

**DOI:** 10.1038/s41746-019-0166-1

**Published:** 2019-09-06

**Authors:** Kit Huckvale, Svetha Venkatesh, Helen Christensen

**Affiliations:** 1 Black Dog Institute, UNSW Sydney, Sydney, NSW Australia; 2 A2I2, Deakin University, Geelong, VIC Australia; 3 Mindgardens Neuroscience Network, Sydney, NSW Australia

**Keywords:** Biomarkers, Psychiatric disorders, Information technology

## Abstract

The use of data generated passively by personal electronic devices, such as smartphones, to measure human function in health and disease has generated significant research interest. Particularly in psychiatry, objective, continuous quantitation using patients’ own devices may result in clinically useful markers that can be used to refine diagnostic processes, tailor treatment choices, improve condition monitoring for actionable outcomes, such as early signs of relapse, and develop new intervention models. If a principal goal for digital phenotyping is clinical improvement, research needs to attend now to factors that will help or hinder future clinical adoption. We identify four opportunities for research directed toward this goal: exploring intermediate outcomes and underlying disease mechanisms; focusing on purposes that are likely to be used in clinical practice; anticipating quality and safety barriers to adoption; and exploring the potential for digital personalized medicine arising from the integration of digital phenotyping and digital interventions. Clinical relevance also means explicitly addressing consumer needs, preferences, and acceptability as the ultimate users of digital phenotyping interventions. There is a risk that, without such considerations, the potential benefits of digital phenotyping are delayed or not realized because approaches that are feasible for application in healthcare, and the evidence required to support clinical commissioning, are not developed. Practical steps to accelerate this research agenda include the further development of digital phenotyping technology platforms focusing on scalability and equity, establishing shared data repositories and common data standards, and fostering multidisciplinary collaborations between clinical stakeholders (including patients), computer scientists, and researchers.

## Introduction

Digital phenotyping^1^ (or personal sensing^2^) is the moment-by-moment, in situ quantification of the individual-level human phenotype using data from personal digital devices. It seeks to exploit the potential of data that are automatically generated and aggregated by smartphones, wearables and other connected devices to measure (or offer robust proxies for) human behavior and function in both health and disease. Today, these data streams include sensor measurements, activity logs and user-generated content.^3^

Data-driven, objective measurement of individual function is of specific interest in psychiatry, which has previously relied almost exclusively on self-reports of mental health symptoms, which has few biological markers, and where diagnostic categories remain unclear.^4–6^ Building on the widespread adoption of smartphones as the principal enabling technology, digital phenotyping has been enthusiastically adopted as a research theme in mental health. Our searches identified over 80 peer-reviewed publications since 2015 that focus on digital phenotyping for psychiatric conditions.

Many of these studies appear to anticipate that digital phenotyping should play a role in routine clinical practice, for example by enhancing aspects of clinical diagnosis and treatment through earlier detection of condition onset, relapse or treatment response. As a result, there is a timely opportunity to consider what this vision of clinical digital phenotyping might require in terms of scope, quality and safety in order to be used in practice. Three factors motivate this question. The first is the historically slow pace of translation of health innovations into practice. Reported lag times of 17 years^7^ are at least partially accounted for by mismatches between the outputs of research and what—in terms of both design and supporting information^8^—is needed for adoption. The second, relatedly, is the formalization of approaches to health technology assessment which act to codify criteria for adoption, such as cost-effectiveness.^9^ The third acknowledges the risk posed by technology change: should it take 17 years to find practical uses for digital phenotyping, it may well be that the underlying technologies are obsolete. There is a risk that, without such consideration, the potential benefits of digital phenotyping are delayed or not realized because clinically-feasible approaches, and the evidence required to support clinical commissioning, are not generated through timely research.

The purpose of this review is to highlight how developments in digital phenotyping have created a broad range of potential clinical uses, to identify salient gaps, and to highlight opportunities for action intended to promote future clinical adoption, quality and safety (summarized in Table 1). The review argues that a range of developments are needed to accelerate progress, which include the development of scalable data collection infrastructure that addresses equity and privacy issues, the application of methods from machine learning to process and analyze signals, and the development of validation approaches for data quality that address challenges of bias and noise inherent in population-scale phenotypic data. We also highlight the potential clinical value of integration between digital phenotyping and digital interventions in order to accelerate adoption.

**Table 1:** Seven priorities: opportunities and practical steps for progressing a vision of clinical digital phenotyping

#	Priority
1	Applying digital phenotyping to the mechanisms and behaviors underlying psychiatric disorders rather than outcomes alone.
2	Prioritizing research into digital phenotyping according to realistic clinical uses.
3	Anticipating clinical quality, safety and acceptability issues that will act as barriers to implementation and uptake.
4	Combining digital phenotyping with digital interventions.
5	Developing data collection platforms with a focus on issues of equity, trust and privacy.
6	Developing shared data resources to accelerate collaborative research, replication and scale-up studies.
7	Establishing strong collaborations with healthcare professionals, providers and computer science.

Reflecting the topic of this special collection, we draw examples from mental health in general, and adolescents and young adults, specifically. This group represents a potentially important target group for mental health-focused digital phenotyping. Both the incidence and overall prevalence of serious mental disorders peaks in those aged 18–30. Unlike younger children who often do not yet have a personal smartphone, device ownership is ubiquitous, and—in Australia—higher in this group than in any other demographic. Nevertheless, the opportunities we identify are not restricted to this age group or condition area and are of potential relevance in any clinical domain where digital phenotyping is being considered.

### Mapping digital phenotyping to potential clinical applications: examples from youth mental health

There are now a broad range of potential applications for digital phenotyping with clinical relevance in youth mental health and that are the subject of active evaluation. These span the breadth of care stages, from screening, diagnosis, monitoring, and treatment, including early intervention and relapse prevention. These are summarized in Table 2 and discussed below. (Because the primary youth literature is limited, we also include examples of digital phenotyping that are relevant in youth either because of the developmental significance of the condition or because the peak age of onset occurs in this age group.)

**Table 2:** Spectrum of youth-relevant mental health applications being explored using digital phenotyping

Prevention	Screening and early diagnosis	Monitoring	Treatment
Fostering resilience and health promoting behaviors	Proactive identification of undiagnosed conditions and/or formal confirmation of a specific condition	Early detection of condition changes, adverse events, and relapse	Tailored intervention, engagement and treatment efficacy monitoring
**Stress identification** ^13– 17^ Passive detection of changes in self-perceived stress in order to foster self-regulation and resilience or trigger proactive help-seeking before the onset of frank mental health symptoms. **High-risk alcohol use detection** ^49, 50^ Passive identification of high-risk drinking episodes using activity and phone utilization data in order to trigger prevention interventions.	**Mood disorder detection** ^10– 12^ Passive detection of activity changes using accelerometry, GPS, phone utilization data in order to identify individuals at risk for depression or anxiety. **Suicidality detection** ^51– 53^ Automatic natural language processing of social media posts to identify at-risk individuals.	**Mood disorder self-monitoring** ^19^ Using activity and location data to discreetly monitor mood changes as part of combined parent-child self-monitoring intervention. **BPD relapse prediction** ^12, 23, 26– 29, 37, 38^ Passive monitoring for depressive (using keyboard signals) and manic (using voice signals) signs indicative of relapse, enabling “early warning sign” interventions. **Opioid overdose detection** ^40^ Active abnormal respiratory pattern detection post opioid use using smartphone “sonar” (combining speaker and microphone.) **Schizophrenia relapse prediction** ^124^ Passive monitoring for early-warning signs using accelerometry and heart variability in order to detect relapse early and enable medical intervention.	**Depression therapy enhancement** ^22, 112^ Sensor-derived signals (e.g. location information) used to tailor therapy in order to maximize user engagement and treatment effect or identify when treatment is not working.

*BPD* bipolar disorder

#### Mood disorder identification, tracking, and predicting subsequent treatment response

Within student and young adults cohorts, passive detection of activity changes using accelerometry, GPS, and phone utilization data has shown promise for identifying individuals at risk for self-reported depression or anxiety^10–12^ as well as potential upstream determinants of future mental ill health, such as self-reported stress.^13–17^ Although mood disorders are most commonly clinically diagnosed between the ages of 25 and 30,^18^ it is increasingly recognized that psychological distress may predate this by many years, either as subclinical symptoms or because of delayed help seeking relating to stigma and poor expectations of clinical support. Digital phenotyping strategies that can identify these at-risk and un-diagnosed individuals might offer a way to alleviate significant morbidity and future clinical demand, as well assist parental and self-monitoring in adolescents once diagnosis is confirmed.^19^ Further work is required, however, to elucidate the relationship between population scale measurement of constructs such as “stress” that are operationalized in different ways and the ultimate development of mood disorders.

Separately, an important potential opportunity in the clinical management of depression is predicting treatment response.^20^ Only 50% of individuals respond to the first treatment they are offered,^21^ and lengthy trial-and-error approaches incur significant patient and health-service costs. Digital phenotyping using voice analysis^22^ provides a proof of concept for new methods to predict treatment response but improvements in prediction accuracy are now needed to enable clinical uses.

#### Bipolar disorder and relapse prevention

In bipolar disorder (BPD), there has been substantial progress in the development of digital-phenotyping techniques for condition monitoring and relapse detection. Changes in location and activity patterns,^12,23–25^ keyboard interaction dynamics,^26,27^ social phone utilization metrics^24,25,28^ (such as calls placed and received) and voice^26,29^ (for example, captured from phone calls) have been used, alongside active measures, to predict both manic and depressive states. Relapse is common in BPD, with 70% of individuals experiencing deterioration or recurrence within5 years of a manic episode.^30^ Despite subtle symptoms routinely being present at up to 4 weeks^31^ before relapse, access to timely treatment remains a major issue, partly because symptoms can be highly patient-specific^31^ and partly because comorbid factors, such as drug use and co-existing psychiatric disease,^32^ affect the capacity of individuals to respond effectively. Early-warning sign interventions that rely on self-monitoring are desirable for young adult BPD patients^33^ and effective in increasing time-to-recurrence while reducing hospitalisation.^34^ The development of digital phenotyping-based methods promises early warning sign services that could be offered to individuals who would otherwise find it hard to sustain self-monitoring.^28,35,36^ The first randomized^37^ and cohort^37^ studies are now either underway or will start shortly.

#### Opioid overdose detection and harm reduction

Opioid overdose carries a high risk of respiratory failure and death. In 2016, 245 Australians aged 15–34 died of opioid overdose, of which 216 (88%) were accidental.^38^ This represents a 31% increase in yearly deaths compared to a decade earlier (6.98 deaths per 100,000 in 2016 compared to 5.33 in 2006). Opioid toxicity is reliably treatable using the drug naloxone if caught in time, but users often struggle to identify signs. A smartphone-based, harm-reduction solution that uses digital phenotyping to detect signs of respiratory distress^39^ raises the prospect of reducing accidental overdoses by, for example, contacting community first responders to administer naloxone^40^ or prioritizing those with detected near-overdoses for methadone therapy in order to avoid future events.^41^

#### Detection of harmful alcohol drinking behaviors and exposures to alcohol-related messaging

Some of the only phenotyping literature that focusses explicitly on a youth population has explored whether alcohol-related exposures can be predicted using passive monitoring of location data^42–47^ and, separately, if alcohol consumption behaviors can be predicted using smartphone sensing and activity data.^48,49^ These uses highlight how digital phenotyping can also be used for public health purposes by generating information not only about individuals but concerning aggregate patterns of behavior that can then be used to inform structural interventions, for example, ensuring that retailers are complying with the law in relation to the supply of alcohol to minors in locations where problem drinking emerges as a pattern from digital data.

#### Identification of risk of suicide in the wild

Automatic natural language processing of social media posts has been used successfully to identify individuals with evidence of psychological distress^50–52^ that might place them at risk of self-harm or suicide. Suicide is a leading cause of death amongst children and young people,^53^ and predicting rapid escalations in the risk of suicide is a policy priority, particularly given the development of new, effective interventions, such as ketamine for rapid reduction of depressive and suicidal symptoms.^54^ Proactive screening in online environments raises important privacy questions but recognizes that many suicides occur out of the blue, prior to contact with health professions. Within at-risk populations, signals from smartphones^55^ and clinical measurements (such as electrocardiography to detect heart rate variability^56^) may offer a discreet way to provide a safety net.

### Gaps and opportunities

Despite the potential described above, today only a few research and healthcare organizations are collecting digital signals, and these activities are largely exploratory. The data that result are small-scale (typically involving a few tens of people monitored for only a short period of time^36^), partial, unstandardized and often not linkable, resulting in multiple, small data “silos”.^57^ These are not suitable for robust identification of digital biomarkers concerning mental illness onset, treatment response or relapse. They are also often insufficient for effective analysis (for example, beyond simple correlations), because the data are small-scale and noisy. The development and application of appropriate methods for study execution and the analysis of digital phenotyping data has already been identified as a priority for the future clinical relevance of the field.^58^ Sitting alongside this, we perceive several additional opportunities that collectively stand to accelerate the clinical impacts of digital phenotyping.

### Opportunity 1: applying digital phenotyping to the mechanisms and behaviors underlying psychiatric disorders rather than outcomes alone

An emerging template for contemporary studies in digital phenotyping is to explore through correlations,^59,60^ modelling^61,62^ or machine learning,^17,63^ the relationship between a set of smartphone-derived sensor and utilization features and the result of a self-completed outcomes instrument, such as the PHQ-9 for depression. This approach has yielded new, clinically-relevant phenomena, such apparent changes in smartphone-measured sleep continuity^64^ and location-based activity^65^ that precede a major depressive episode by weeks and could therefore have potential uses for onset and relapse prediction. This kind of data-led, clinical endpoint-based approach recognizes that there are a large number of potential sensor and analytics data sources, each of which may be (at least in advance) of uncertain significance in relation to a given clinical outcome, as well as being amenable to any number of summary representations (for example, due to complex temporality and missingness.^66^) Nevertheless, we want to highlight the potential value—both for clinical applications and research—of a complementary, mechanistic approach that considers not only clinical outcomes but also intermediate functional and behavioral states,^2^ as well as disease-related processes, as potential targets for prediction using digital phenotyping.

Consider, for example, the relationship between cognitive dysfunction and depression, which typically presents first in early adulthood.^18^ Subjective impairment of thought and concentration forms part of the diagnostic criteria for major depressive disorder,^67^ while objective testing consistently identifies a range of specific functional deficits in executive function (including processing speed), memory and attention.^68–70^ These deficits are present at initial diagnosis^71,72^ and have been identified as potential trait markers for depression^73^ in at least a subset of individuals.^72^ It is already known that cognitive dysfunction improves with therapy and may predict specific treatment response,^74,75^ such as the likely success of talking therapies, disease course^72^ and future neurodegenerative illness.^76^

This example illustrates firstly how clinically useful opportunities can exist for measuring specific facets of a disorder rather than its overall state using digital phenotyping. Being able to quantitate cognitive change using a phenotyping-based approach is attractive because current psychometric and neuropsychiatric tests rarely reflect specific cognitive processes unambiguously, can be unwieldy, take time, and are poorly standardised.^73^ Yet, because cognitive function may predict treatment response,^21^ it is attractive as a target for prediction, particularly given the resource and patient costs associated with the selection of ineffective therapy.^77^ The research goal here should be to identify proxies for cognitive tests which are practical to apply quickly and routinely, and which offer timely and more precise signals of improvement.

Secondly, the causal and temporal relationships between cognitive dysfunction and affective symptoms is itself an open research question that is amenable to exploration using phenotyping.^73,76^ The potential feasibility of discreet, continuous digital phenotyping in young adults offers a route to address the specific call for longitudinal studies^73^ that can assess if and how cognitive symptoms precede the peak onset of depression in the mid-late twenties.^18^ Because depression state accounts for a only small proportion of the observed variation in cognitive function between individuals,^68^ the capacity of digital phenotyping to capture detailed within-individual data^66^ is also important.

Thirdly, this focus can act as a rational guide as to what signals are captured from users’ digital behavior in the first place. For example, the observation that specific sub-measures of executive function, such as semantic and phonemic fluency, are significantly reduced in first episode depression^72^ reasonably directs attention to device activities where these capabilities might be exercised, such as word-finding whilst typing. In BPD, similar metrics have yielded new potential cognitive markers.^26^ Effort can then be directed to the feasibility of collecting these data, for example the technical ability to monitor a user’s on-screen keyboard versus understandable potential privacy concerns.

This guided approach is important not only because there is otherwise a large space of things that could be measured, but because it is becoming clear that digital phenotyping is not always optimal for the detection of particular behavioral signals. For example, sleep detection using smartphones overestimates sleep duration and underestimates sleep disturbance compared to formal actigraphy,^78^ while self-reported mood using experience sampling methods substantially improves model prediction compared to digital phenotyping alone in depression.^63^ Having reference standards (such as validated functional measurement instruments) that are conceptually “closer” to the original data sources should make it easier to critically select, assess and refine what is used for modelling. Because each measurement comes with a concrete cost both in terms of implementation and user experience (e.g. in battery life impact, data transfer costs and/or perceived impacts on privacy or acceptability) a rational approach to selection may also help to avoid wasted effort.

The mechanistic approach we describe here emphasizes the value of the existing physiological measurement and psychometric literature in suggesting intermediate targets for proxy prediction by digital phenotyping. We are not new in promoting this type of strategy.^2,79,80^ For example, Mohr and colleagues advocate using digital phenotyping to build markers of behavioral traits and use these, in turn, to explore relationships with higher-level states.^2^ Our approach is complementary in advocating a focus on signs and symptoms with established (or emerging) direct practical clinical use. Importantly, neither precludes using these intermediate targets in turn to predict clinical endpoints. It is an empirical question as to whether models built in this way will have greater predictive power than those that attempt to link raw data directly to outcomes, and future work should critically assess this potential.

### Opportunity 2: prioritizing research into digital phenotyping according to realistic clinical uses

Digital phenotyping strategies, as with any health technology intended for clinical use, will ultimately need to demonstrate both efficacy and cost-effectiveness. What is acceptable performance, however, is substantially contingent on the intended clinical application. An illustrative comparison can be made for BPD between screening, e.g. for detecting onset in early adulthood,^12,81^ and monitoring existing patients, e.g. for relapse detection.^28^

One argument made in favor of digital phenotyping is that the ubiquity of consumer digital devices will enhance the reach of the services that result.^5,58^ From the point of view of the technical performance of digital phenotyping-based screening for new conditions, however, a population-focused strategy cannot escape the challenges that apply to any screening programme.^82^ For example, assuming a point prevalence for clinical BPD of 0.6% in adolescents,^83^ any new digital phenotyping screening test would need to have a specificity of at least 99.4% (assuming perfect sensitivity, and no targeting other than by age) if the group of those who positive is not to be dominated by false positives. Even assuming an appetite for false positives, representative of existing clinical tests,^84^ that allows for 10 false alerts for every true case, a specificity of 94.0% is required. (For context, the best specificity of the relevant BPD studies we reviewed was 87.2%.^12^) Specificity problems may be enough to discount a screening test, given the dual burden of unnecessary worry in a non-clinical population and actual healthcare costs associated with managing people who present incorrectly as screen positives. Parallel issues affect sensitivity, particularly when differences between healthy and diseased populations are small or where measured changes account for only a small proportion of inter-individual variance.^70^ These issues are not simply theoretical. Commercial digital phenotyping platforms whose stated purpose is to support population-scale diagnostic screening are already being piloted in health systems despite unclear evidence concerning their psychometric properties.^85,86^

By contrast, specificity may be less of concern for monitoring of those with an established condition population for signs of relapse. The absolute numbers involved are likely be smaller, limiting the scope for burden on service delivery. Individuals may find a false positive risk acceptable if this means that genuine episodes are not missed. And, importantly, these trade-offs can be explicitly stated in advance so that individuals can make an informed choice. Finally, established caring relationships might mean that false positives can be more efficiently triaged out (based on the known profile of each patient, particularly if digital phenotyping can be paired with continuous self-monitoring) without excessive cost or distress. In this scenario, rather than maximizing specificity, it may well be that sensitivity becomes the most important issue given the costs and burden associated with an unmanaged relapse.

Technical test performance is not the only relevant concern. Established principles for clinical screening programmes^82^ require, for example, that conditions have a prodromal phase in which early intervention yields clinical benefit. Despite interest in digital phenotyping for Parkinson’s Disease (PD) that focuses on motor symptoms,^87^ early therapy is not clearly beneficial for the control of these symptoms.^88^ Similarly, for depression the clinical significance in terms of future progression to depression of subclinical low mood in otherwise healthy individuals—particularly at a population scale—is unclear. Importantly, this does not imply that these approaches lack value. For example, a digital phenotyping test deployed prospectively to those who present with motor symptoms could offer a low-cost aid to diagnosis (or subtype elucidation) in PD, which is frequently mis-diagnosed.^89–91^ Focus could also be directed towards targets that are modifiable. Unlike motor symptoms, cognitive impairment, which is prevalent in PD, appears to respond to targeted intervention, for example using exercise.^92^ Digital phenotyping used to either detect or monitor cognitive changes could usefully inform rational clinical management in early disease (when intensive clinical monitoring is otherwise not warranted.) Prevention strategies may also be more tolerant of false positive results if the follow-on intervention is simple, low-cost and generally acceptable. Many digital interventions could fall into this category of response.

The differences between the two scenarios outlined at the start of this section (both ostensibly using digital phenotyping as a diagnostic test for a defined change in health status) underline how the choice of application shapes not only the risks and potential costs involved but the standards to which tests should be held. While it is not unreasonable to assume that there will be improvements in classification performance as the field develops, and while calls for larger scale studies^36^ (which promise better performance) are timely, a clear sense of the ultimate clinical goals remains important to gauge progress. In our view, there is no reason to delay this. For some applications, it will not be possible to achieve diagnostic accuracy statistics of 99% or higher. In order to avoid wasting effort and time, and to ensure that the evidence base needed to support clinical commissioning develops effectively, consideration of how clinical priorities (whether on grounds of burden of disease, potential resource saving or unmet need) intersect with technical feasibility should be a routine feature of research goal-setting now. Without this, there is a risk of outputs that have no realistic prospect of being used in clinical practice. At the very least, these test accuracy statistics should be fully reported; it is not uncommon to see sensitivity (recall) and the positive predictive value (precision) being reported, but not specificity. Study authors may reasonably contend that the balance of these issues will vary according to setting, provider risk appetite and patient attitudes. Decision frameworks, such as net benefit,^84^ offer researchers a way to model tradeoffs between costs-harms under a range of clinically realistic scenarios without having to commit to a particular solution. These kinds of models should be routinely reported in digital phenotyping studies.

### Opportunity 3: anticipating clinical quality, safety and acceptability issues that will act as barriers to implementation and uptake

Implementation-relevant concepts of quality and safety are well-operationalized in clinical medicine, for example as the six Institute of Medicine quality domains.^93^ These span safety, effectiveness, patient-centredness, timeliness, efficiency and equity. While each is relevant from the point of future implementation of digital phenotyping, several dimensions are salient.

#### Person-centered care

Because patients and consumers are the ultimate gatekeepers of whether it is used, clinical digital phenotyping will rely on a person-centered approach. For complex long-term conditions, there are benefits in monitoring strategies that moderate treatment burden^94^ by reducing explicit self-monitoring and the constant reminders of health status and functional limitations that this can entail. Conversely, some groups may prefer the active engagement that self- or professionally-supported care entails. For example, a qualitative study of potential young adult users of app-based behavior change interventions found that most were not receptive to the use of contextual tailoring, of the kind that digital sensing could provide, to augment these tools.^95^ Beyond individual preferences, the potential for consequential impacts on self-management and self-regulation skills of increasingly automated measurement remains an open question.

Digital phenotyping relies, both in development and subsequent application, on the ongoing willingness of users to grant access to the various data streams, from on-device sensors to third party social media, that provide insights into their daily behavior. There are multiple potential trade-offs that patients and the public might want to consider in making an informed choice about whether to consent to pervasive monitoring.^96^ Privacy and data governance are understandable topical concerns, given the repeated identification of poor privacy practices by large internet companies and repeated failures observed in related consumer technologies, such health apps.^97^ The development of digital phenotyping entails technical choices including where, for example, data will be processed and stored—particularly if machine learning models are cloud-based—with real potential implications on the acceptability of the ultimate solution to users. In addition, although more trusting of doctors than other groups, patients appear to be way of sharing certain types of data, such as location, which is routinely used in digital phenotyping.^98^ Strategies will therefore be needed to empower providers to negotiate appropriate access to these data.

How these trade-offs play out will inform the feasibility of different digital phenotyping approaches. For example, individuals with a serious mental health issue may be highly motivated to avoid the risk of relapse even if this requires extensive data about everyday life, including sources such as voice samples.^29^ There is an opportunity for user-centered research that explores the detail of these compromises.^98,99^ This should seek to focus effort towards applications that are likely to be acceptable to both patients and clinicians (and therefore actually usable), to identify effective strategies for supporting individuals in making informed choices about digital phenotyping without the risk of coercion, and in identifying user “red lines” (for example, about how data will be handled) that have practical implications for the design and cost of the technology platforms that underpin digital phenotyping.

#### Equity

Equity is a relevant issue in digital phenotyping for at least three reasons. First, there is a risk of excluding groups of users if underlying technology development favors certain commercial platforms or is predicated on sensing or other technologies only available in latest generation devices. For example, a majority of studies to date collect data using the Android mobile device operating system, reflecting technical challenges in enabling reliable continuous sensing on Apple devices.^36^ In Australia, however, for example, Apple devices account for over half of mobile market share.^100^ Addressing this disparity should be a priority, therefore. There is a strategic opportunity for the research community to proactively engage with Apple and Google not only to address salient technical challenges but also to ensure that digital phenotyping is understood as a valid (and valued) use case. Without this kind of engagement, there is a risk that unforeseen changes to privacy rules or platform software will unexpectedly disrupt the function of digital phenotyping apps.

A second issue relates to the use of machine learning as a foundational technology for translating digital phenotyping signals into usable information. Machine learning models trained in limited populations (such as college students) can demonstrate unacceptable bias in real-world applications (a problem known as “distributional shift”^101^), such as image classifiers trained on majority white populations that consistently fail in other groups.^102^ Selection bias has already been identified as a potential risk in digital phenotyping studies of BPD.^36^ Consequently, digital phenotyping applications that incorporate machine learning need to attend to evolving standards and evaluation methods designed to assure fairness in clinical machine learning.^103^ These include, for example, ensuring that test/training populations have the same distributional characteristics as the populations in which the digital phenotyping will be used, attending specifically to model performance in ‘protected groups’ who represent minorities and those historically subject to inequity, and considering the potential for digital phenotyping to reinforce existing subtle biases in the clinical management of patients.^103^ One immediate consequence for digital phenotyping research is to challenge the assumption that convenience samples are routinely good enough to condition models.

Finally, and relatedly, assumptions about how users interact with their devices may not be valid for different user groups. For example, in our experience, many adolescents have limited mobile data allowances that limit the potential for bulk data collection. Because the activation of device sensors is associated with additional power demands, limited battery capacity—or the ability to charge devices on demand—may also be relevant, for example in homeless youth.^104^ Similarly, the assumption that smartphones are “always carried and always on” may not be valid in older populations, limiting their ability to derive value from digital phenotyping strategies that rely on continuous signals. Recognizing that there may be constraints associated with specific populations does not mean that nothing can be done. For example, in youth and other populations who are sensitive to cellular data costs, it may be feasible to configure digital phenotyping apps that wait for the availability of a wireless (i.e. no cost) data connection before attempting to transmit data for analysis. Alternatively, where data latency needs to be controlled, it may be appropriate to offer resource to covers the cost of cellular connections. Where device energy concerns exist, it may be feasible to selectively activate sensors, for example in response to contextual triggers, to reduce the total impact on battery life. There is also an opportunity for future digital phenotyping analyses to routinely model the minimal data required to generate usable signal.

#### Efficiency and safety

Particularly in primary care, where practitioner time and resources are constrained, it is imperative that digital phenotyping strategies can be effectively integrated into clinical workflows. This means understanding early the value that healthcare professionals will attach to different forms of information arising from digital phenotyping and anticipating practical concerns such as clinical systems integration – and upstream requirements, such as certification and data standardization.

A related concern is the validation and safety assurance process for algorithms intended for clinical use, particularly where these are based on machine learning techniques that may have subtle, hard-to-anticipate failure modes.^101^ The development of standardized approaches for documenting digital phenotyping strategies, including machine learning feature and algorithm definitions, is an open opportunity, as are approaches to validation and testing that can reliably uncover safety-relevant issues.

### Opportunity 4: combining digital phenotyping with digital interventions

Digital phenotyping is anticipated to create clinical value through “closing the loop” between detecting clinical phenomena and taking action by using signals to trigger, tailor and deliver personalized digital treatment or prevention interventions.^5^ This is particularly relevant to psychiatry, where the development and adoption of both personalized treatments and digital interventions is a priority.^105^ Digital interventions can incorporate health promotion, lifestyle education, and psychological therapies, and have a proven record in the treatment and prevention of depression and anxiety,^106^ smoking cessation^107^ and for the management of diabetes,^108^ asthma^109^ and cardiovascular disease.^110^ Contextually enhanced eHealth interventions that tailor advice and guidance to the setting and experiences of individuals^111,112^ offer a potential avenue to reduce treatment costs^113^ while addressing the challenge of poor adherence seen with current digital interventions.^114^

**Fig. 1:**
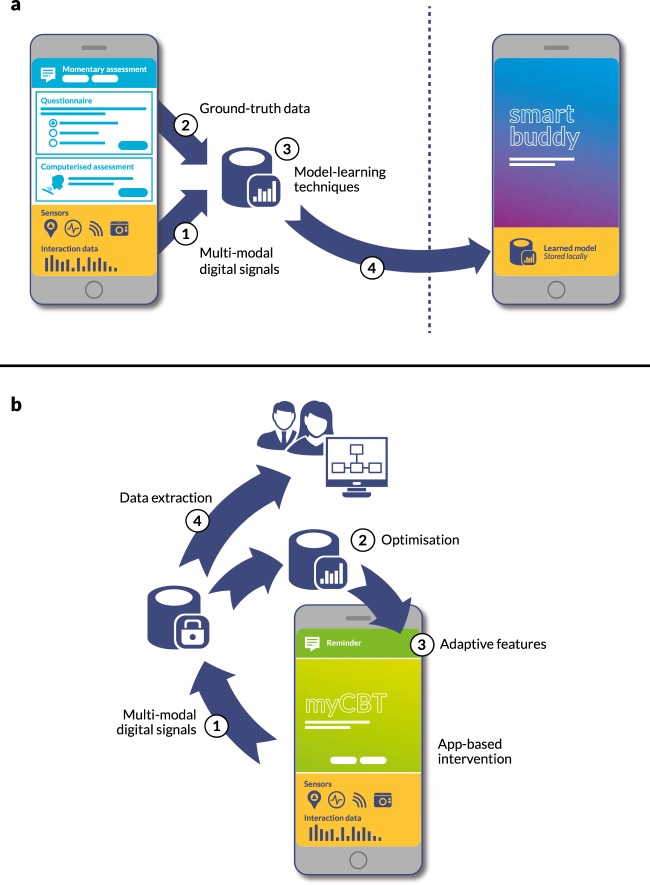
Two models of integration between digital phenotyping and digital interventions. Figures and letters refer to those shown in the diagram. Model (A) describes a “learn-then-implement” approach where (1) multi-modal digital signals (e.g. sensor data) are combined with (2) ground-truth data (such as self-reported mental health) and used to learn a digital phenotyping predictive model, for example, predicting a change in mental health status from GPS and activity data. This model can then be deployed into future interventions (4) to trigger intervention components based on changes in mental health state predicted by digital signals alone. Model (B) describes a “continuous learning” approach, where (1) digital signals are automatically collected alongside intervention outcomes data. These are used to (2) continuously update and refine an intervention model conditioned on some goal, for example achieving a positive change in mental health status. This model is then used to trigger and tailor different aspects of the intervention (3). The resultant outcomes feed back into the learning process. Data collected via this approach can also be extracted for analysis (4)

Many existing digital phenotyping applications appear to be already intervention-like, for example integrating experience sampling as a principal data source and summarizing longitudinal data used for modelling and prediction in ways that are intended to be accessible to users. The need to package phenotyping within an app wrapper for deployment to users’ smartphones creates an obvious context to extend this with intervention content that is tailored and responsive to the signals generated through digital biomarkers in order to return value to users. There are multiple ways in which this could be achieved. For example, models resulting from digital phenotyping studies could simply be integrated into future interventions and used, for example, to trigger contextual intervention content (Fig. 1a). Or, alternatively, digital phenotyping data could be used to drive online optimization,^115^ where intervention tailoring models are continuously updated (Fig. 1b).

Enhancing self-management in this way is a potentially good fit with stepped care approaches, such as those now established in the management of mood disorders,^116^ where objective data-driven guidance can be used to enhance the capacity of individuals to effectively self-manage early-stage and less severe illness. It offers a route to maximize the potential value that can be extracted from the large amounts of data that are necessary to drive digital phenotyping by creating a context where data can be visualized and used for structured self-reflection with a defined therapeutic purpose. In addition, the use of digital sensing data for features such as tailoring and soft recommendations—rather than in formal diagnostic or therapeutic processes—may be more realistic in terms of safety and technical feasibility. Because within-individual variation appears to be an effective predictor of condition onset or change in mental health,^66^ closed loop interventions may be particularly valuable here. For example, future approaches could leverage Bayesian optimization^117^ to build n-of-1 predictive models tailored to the specific user. These closed-loop systems offer a new context to explore mechanisms and trajectories of illness development and treatment response. In addition, integration with digital interventions may itself create entirely new opportunities for digital phenotyping, for example, using automatically collected data about interactions with the intervention itself to generate ‘engagement phenotypes’ that can be subsequently used for tailoring.

### Next steps

In order to respond effectively to the opportunities identified above, practical and coordinated action stands to help accelerate both research and the ultimate development of real-world health applications for digital phenotyping.

#### Development of shared platforms for data collection

One of the catalysts for digital phenotyping has been the research-led development of sophisticated open technology platforms such as Beiwe,^1^ Purple Robot^62^ and Monsenso.^118^ Reflecting the opportunities identified we have identified above, priorities for the future development of these platforms should include addressing equity concerns by supporting Apple devices, anticipating information and clinical data governance issues as platforms move from research to practical uses (for example, ensuring that cloud-based data processing is within compatible jurisdictions), incorporating features that can expedite data validation and quality assurance, and supporting future integration with digital interventions.

#### Development of shared data repositories

Digital phenotyping studies typically generate rich datasets^64^ which may be exploited for multiple analytical purposes. As a result, there is an opportunity to consider how these might be structured as reusable resources. For example, the development of biobanks has resulted in large numbers of research publications, clarity around researcher market needs and rapid technology development. UK Biobank,^119,120^ opened to research in 2012, provides a case study of how a single, well-designed, public resource can make a significant scientific contribution. By 2018, our bibliometric analysis suggests that research studies using UK Biobank represented nearly a tenth (9.2%, *n* = 462/1727) of annual global biobank-related publications. Of the biobank studies published in the fifty most important clinical and general science journals in 2018, well over half (57%, *n* = 77/120) used data from UK Biobank.

**Fig. 2:**
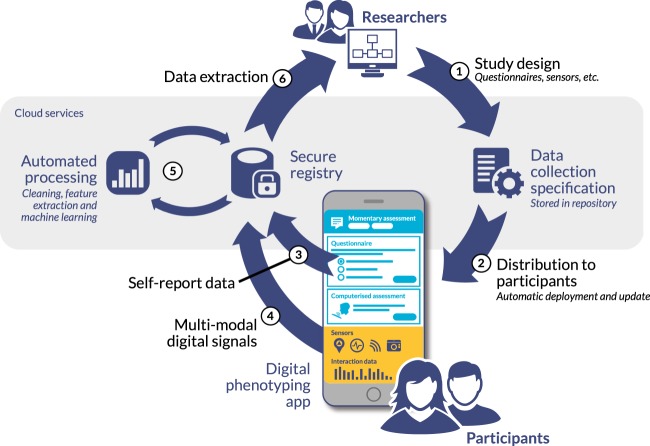
Black Dog Institute/Deakin model for a scalable, integrated multi-user platform for digital phenotyping research Figures and letters refer to those shown in the diagram. In this model, (1) researchers specify the study design, define which questionnaires and sensors are required to deliver a digital phenotyping study (and optionally how these are integrated with any intervention components, such as self-guided therapy.) This specification is then hosted alongside others in a secure online repository. When each study commences, the specification is automatically downloaded (2) to users’ devices by a digital phenotyping app. This app can be a multi-study coordination tool that acts to coordinate data collection, a bespoke, study-specific data collection app, or a hybrid data collection intervention. Collected (3) self-report (e.g. questionnaires and momentary assessments) and (4) digital data (e.g. sensor measurements and device interaction data) is uploaded automatically to a secure online registry. Platform modules automatically manage potential barriers to data collection, such as user battery life and limited connectivity, through smart scheduling and caching. Automated processing pipeline (5) normalizes and converts raw data into standardized intermediate features and labelled outputs using machine learning. Researchers can start to extract registry data (6) as soon as it is received, accelerating analysis, permitting study designs that involve expert feedback, and allowing any data collection issues to be identified and addressed early in the research process. Rights management enables future researchers to request from users’ access to previously-collected data

For digital phenotyping, potential benefits include avoiding duplication of effort, accelerating research, opening the field to a wider range of researchers beyond those already invested in digital health, and being able to pool datasets to tackle issues of statistical power and heterogeneity. Acknowledging topical interest in replicability in psychiatry, there may be specific value in the collaborative development of data standards. Coordination is relevant not only to how raw telemetry data are persisted, but also ensuring consistent acquisition of metadata that affect analysis (such as originating device types, measurement scale/precision and demographic details), socializing best practice around data cleaning and validation pipelines and, where supervised machine learning is used, documenting feature algorithms so that they can be replicated. Development of shared repositories for digital phenotyping will also require consideration of ethics issues, secure storage, linkage potential and logistical issues of exchange potentially large datasets securely across different jurisdictions. At the simplest level, collaboration could involve the development of working groups that work to harmonize data collection and reporting to expedite replication and scale-up studies. Nevertheless, we think that more significant value will be realized by being able to combine and pool data for reuse. Recognizing this opportunity, in Australia, a multi-center consortium is being assembled to develop a large-scale phenotyping databank. The Black Dog Institute and the Applied Artificial Intelligence Institute (A2I2) at Deakin University are building a hybrid data collection platform and data repository that will permit multiple primary and secondary analysis studies to be conducted on shared infrastructure (Fig. 2).

#### Development of multidisciplinary collaborations involving clinical disciplines, providers, patients, user-centered design, and computer science

The success of digital phenotyping is contingent on hospitals, clinicians and health companies wanting to participate in the development of useful products for their patients and health care organizations. There is an opportunity for researchers to engage with these stakeholders to better understand their priorities and needs in relation to digital phenotyping.

Equally, the willingness of patients and the public to adopt digital phenotyping technologies should not be assumed.^121^ Topical user-centered research questions include what expectations different user groups hold about when and how the clinical information that digital phenotyping generates should be returned to them; what ways of presenting, summarizing and guiding appropriate self-management exist that can create genuine value for users; and how different groups weight the potential trade-offs between intrusiveness and personal health value. Given both the rapid evolution of privacy issues affecting consumer technologies and the litany of recent high profile commercial privacy breaches, finding ways to substantively represent the views of patients and the public on an ongoing basis should be seen as a strategic priority, not only to understand the boundaries of what kinds of information can be consumed by digital phenotyping but to assure that community consent exist to develop the field in the first place.

There is also a specific need to work alongside the computer science community to ensure that digital phenotyping research continues to benefit from the latest developments in machine learning, the sub-discipline of computer science concerned with the creation of algorithms and models without relying on explicit programming. Removing the need for human programming is also important for interventions, such as personalized digital therapies involving multiple treatment options, timings, individual preferences and capabilities, where the data space is too complex for humans to easily interpret (or interpret at all) and where the form of good solutions cannot be specified or predicted.^122^ Modelling techniques that can efficiently model intra-individual variation (even with sparse data) now exist and are a promising candidate for analyzing personal longitudinal tracking data. The development of explainer mechanisms and layered models may also offer new ways to interpret how individual signals are integrated into predicting clinical phenomena, with relevance for mechanistic insights into the development and evolution of clinical conditions, such as depression in young people. The opportunity to combine these refined or enhanced phenotype datasets with genetic and imaging data, along with personal, self-report and health information is likely to add value to multiple medical research disciplines and accelerate behavioral health.

## Conclusions

For digital phenotyping to drive benefits in mental health and other clinical domains, serious consideration must be given to the practicalities of future clinical application. To be used, and to be useful, digital phenotyping must fit with established norms of quality and safety, be cost-effective and feasible. The research agenda that responds to these challenges will necessarily be multifaceted and multidisciplinary, spanning consumer and health stakeholder engagement, implementation science, technical development, intervention design and economic evaluation. Importantly, this call should not be interpreted as reducing the value of basic research into mechanistic or technical aspects of digital phenotyping that may not have immediate clinical applications. Nor should it discourage approaches that will necessitate changes to clinical workflows, training or patient experience.

Because many serious mental illnesses first present in youth, and because this group is an enthusiastic adopter of consumer technologies, the successful development of digital phenotyping is of specific relevance to the future, effective care of young people with psychological distress. Only a focused approach will ensure that today’s young people—rather than some future generation—start to realize benefits of improved and better personalized diagnosis, monitoring and intervention.

Equally important is the development of global leadership and collaboration to tackle head on questions of trust and access to data, replicability of findings and capacity-building within clinical workforces for this new science of behavior. Because digital phenotyping stands to address genuine gaps in assessment and treatment of mental health issues, psychiatry is particularly well placed to show leadership in this newest of “big data” disciplines.

## Data Availability

The literature search results that support this narrative review are available from the corresponding author upon reasonable request.
